# The PI3Kδ Inhibitor Idelalisib Inhibits Homing in an in Vitro and in Vivo Model of B ALL

**DOI:** 10.3390/cancers9090121

**Published:** 2017-09-10

**Authors:** Etai Adam, Hye Na Kim, Eun Ji Gang, Caitlin Schnair, Solomon Lee, Solah Lee, Sajad Khazal, Osanna Kosoyan, Marina Konopleva, Chintan Parekh, Deepa Bhojwani, Alan S. Wayne, Hisham Abdel-Azim, Nora Heisterkamp, Yong-Mi Kim

**Affiliations:** 1 Department of Pediatrics, Division of Hematology, Oncology and Blood and Marrow Transplantation, Children’s Hospital Los Angeles, University of Southern California Keck School of Medicine, Los Angeles, CA 90027, USA; etaiadam@gmail.com (E.A.); hyekim@chla.usc.edu (H.N.K.); ejiang@chla.usc.edu (E.J.G.); cschnair@chla.usc.edu (C.S.); solo.lee88@gmail.com (S.L.); solalee@chla.usc.edu (S.L.); sjkhazal@mdanderson.org (S.K.); okosoyan@live.com (O.K.); cparekh@chla.usc.edu (C.P.); dbhojwani@chla.usc.edu (D.B.); awayne@chla.usc.edu (A.S.W.); habdelazim@chla.usc.edu (H.A.-A.); heisterk@usc.edu (N.H.); 2 Department of Leukemia, Division of Cancer Medicine, The University of Texas MD Anderson Cancer Center, Houston, TX 77030, USA; mkonople@mdanderson.org

**Keywords:** ALL, PI3K, drug resistance, CAM-DR, idelalisib, migration, mouse model, leukemia

## Abstract

The quest continues for targeted therapies to reduce the morbidity of chemotherapy and to improve the response of resistant leukemia. Adhesion of acute lymphoblastic leukemia (ALL) cells to bone marrow stromal cells triggers intracellular signals that promote cell-adhesion-mediated drug resistance (CAM-DR). Idelalisib, an U.S. Food and Drug Administration (FDA)-approved PI3Kδ-specific inhibitor has been shown to be effective in CLL in down-regulating p-Akt and prolonging survival in combination with Rituximab; herein we explore the possibility of its use in B ALL and probe the mechanism of action. Primary B ALL in contact with OP9 stromal cells showed increased p-Akt^ser473^. Idelalisib decreased p-Akt in patient samples of ALL with diverse genetic lesions. Addition of idelalisib to vincristine inhibited proliferation when compared to vincristine monotherapy in a subset of samples tested. Idelalisib inhibited ALL migration to SDF-1α in vitro and blocked homing of ALL cells to the bone marrow in vivo. This report tests PI3Kδ inhibitors in a more diverse group of ALL than has been previously reported and is the first published report of idelalisib inhibiting homing of ALL cells to bone marrow. Our data support further pre-clinical evaluation of idelalisib for the therapy of B ALL.

## 1. Introduction

While the survival rates for childhood acute lymphoblastic leukemia (ALL) have improved greatly in recent decades, the survival after relapse remains poor [1] and the toxicity of current regimens [1,2] limits further intensification with additional cytotoxic chemotherapy. The search for targeted therapies to augment existing regimens without overlapping toxicity has included approaches, such as immunotherapy and small molecule inhibitors [2].

Leukemia cells interact with the bone marrow microenvironment through several adhesion molecules. This interaction both provides physical anchoring and generates intracellular signals that regulate proliferation and cell death-these are two of the mechanisms of CAM-DR [3]. The PI3K pathway is a nodal point of a signaling network affecting cell proliferation and survival that is frequently dysregulated in human cancer [4]. PI3K pathway activation by cell surface receptors and adhesion molecules is directly mediated by four class I isoforms of PI3K. The p110δ isoform is critical in leukocytes [5], as mice harboring deleted or mutated PI3Kδ exhibit a B-cell defect with decreased mature B-cell numbers and impaired antibody production. PI3Kδ knockout mice also show reduced Akt phosphorylation (p-Akt) in activated B-cells [6,7].

As such, PI3K signaling has been a target of many inhibitors being developed recently but enthusiasm for targeting this pathway has been diminished because, to date, PI3K-directed therapy has shown only modest single-agent activity [8] and the nature of resistance is not well understood [9].

Idelalisib (CAL-101, GS-1101) is a PI3Kδ-specific inhibitor that is approved by the FDA for treatment of relapsed chronic lymphocytic leukemia (CLL) in combination with rituximab, and as monotherapy in multiply-relapsed follicular lymphoma and small cell lymphoma [7,10,11]. Idelalisib has been shown to have a number of effects on ALL, including down-regulating the PI3K-AKT-mTOR pathway and decreasing leukemia burden in a mouse model of Philadelphia(Ph)-like ALL [12], decreasing growth and cell cycle progression in pre-B ALL cell lines (but not the pro-B or mature B ALL cell lines tested) [13], and inducing apoptosis in a patient sample of relapsed ALL with a TCF3-PBX1 fusion [14]. GS-649443 has similar selectivity for PI3Kδ as idelalisib, but better pharmacokinetics in mice [15,16]. GS-649443 was shown to increase survival in a mouse xenograft model using NALM6, decreasing progression of CNS disease as monotherapy and showing synergy with cytarabine in combination therapy [17]. Both idelalisib and GS-649443 were shown to decrease the migration of NALM-6 to SDF-1α in an in vitro transwell assay [17].

Leukemia cells, like hematopoietic stem cells, are in equilibrium between the bone marrow and circulation in peripheral blood and must have a homing signal to guide them to the bone marrow [18]. Clinical experience with idelalisib in CLL shows that drug treatment commonly leads to lymphocytosis within the first weeks of treatment and this effect has been shown to be due to a redistribution of cells from the lymph nodes to the peripheral blood [19,20]. Pre-clinical studies of idelalisib in CLL demonstrated that the drug decreased phosphorylation of Akt [21]. In CLL, cell adhesion through integrin α4 to endothelial cells’ VCAM-1 results in the phosphorylation of Akt, which was shown to be sensitive to inhibition by idelalisib [20]. These observations raise the possibility that the blocking of microenvironment/extrinsic signals might be relevant to the success of PI3Kδ inhibition using idelalisib in CLL. In the current study, we have examined this in B ALL. We tested both idelalisib and GS-649443 on primary patient samples of several genetic subtypes to determine if PI3Kδ inhibitors can suppress homing of ALL cells mediated by SDF-1α and if they affect p-Akt and proliferation of ALL cells.

## 2. Results

### 2.1. PI3K Isoforms are Expressed in B ALL Cells

**Figure 1:**
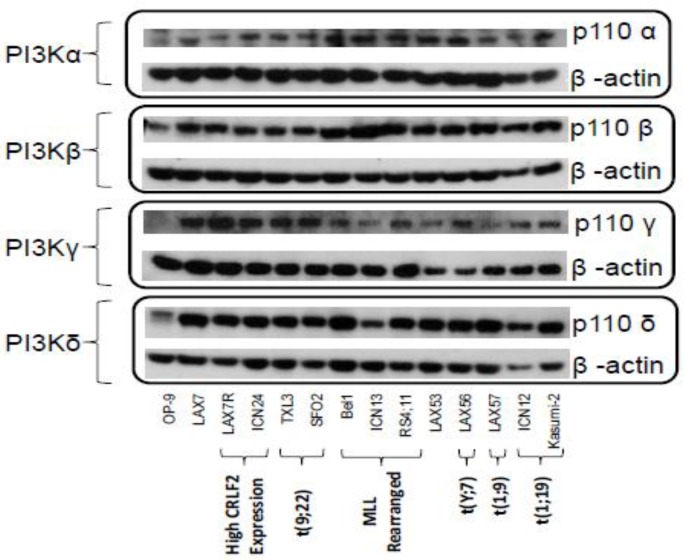
PI3K isoforms in various B ALL cells demonstrated by Western blot (WB). WB performed for p110 subunit of each of the four class I isotypes of PI3K. OP9 cells serve as a co-culture control. Characteristics of each ALL sample are included below the name.

Thirteen B ALL samples containing various genetic lesions (Supplemental Table S1) were analyzed for the protein expression of four PI3K isoforms: α, β, γ, and δ. Murine OP9 stromal cells were used as a negative control for hematopoietic cells. PI3Kδ was identified in all thirteen ALL samples, as were the isoforms PI3Kα and β. PI3Kγ was present in all ALL cells tested, but not in OP9 stromal cells (Figure 1). The presence of PI3Kδ in all of the leukemia samples irrespective of their karyotype supports the prospect of using PI3Kδ inhibition in the treatment of B ALL.

### 2.2. p-Akt^ser473^ Activation by Ligands and Stroma

**Figure 2:**
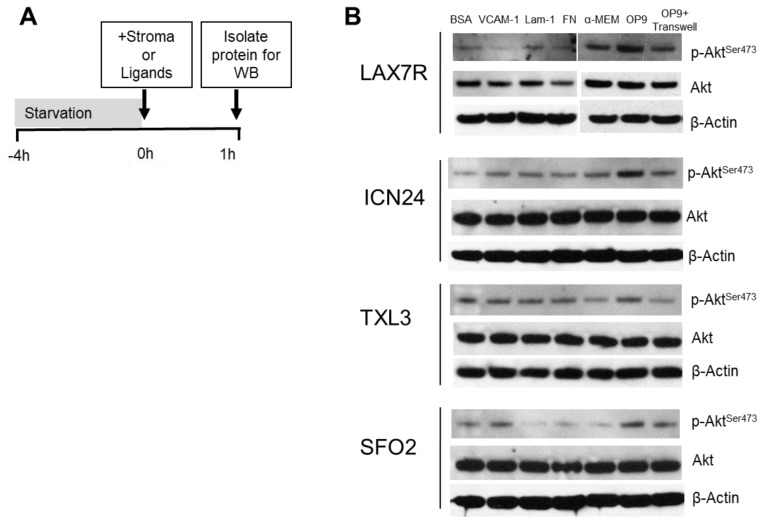
p-Akt activation of B ALL cells by integrin ligands or stromal cells. (**A**) Timeline of starvation and stroma/ligand stimulation assay. Primary B ALL cells LAX7R, ICN24, TXL3, and SFO2 were starved in medium for 4 h, then cultured with plates coated with integrin ligands, stromal cells, or a control. (**B**) Western blot of Akt phosphorylation in these cells.

The PI3K pathway can be activated by a number of extracellular stimuli, including growth factors and engagement of integrins. To test the extent to which PI3K-mediated stimuli can generate p-Akt in different B ALLs, we plated primary patient-derived ALL samples (LAX7R, ICN24, TXL3, and SFO2) and starved them to render them quiescent (Figure 2A). Next they were stimulated with various ligands or direct and indirect stromal cell contact followed by analysis by Western blot for total and p-Akt levels. As shown in Figure 2B, all showed increases in p-Akt after OP9 contact compared to medium control. ICN24 and SFO2 showed an increase in p-Akt when plated in transwells above OP9 cells compared to medium control. The ligands tested showed stimulation of Akt phosphorylation in some samples but no effect in other samples. Taken together, these results show that the stimulation of p-Akt by stromal cells is possibly due to a combination of contact and soluble factors.

### 2.3. Idelalisib Decreases p-Akt^ser473^

**Figure 3:**
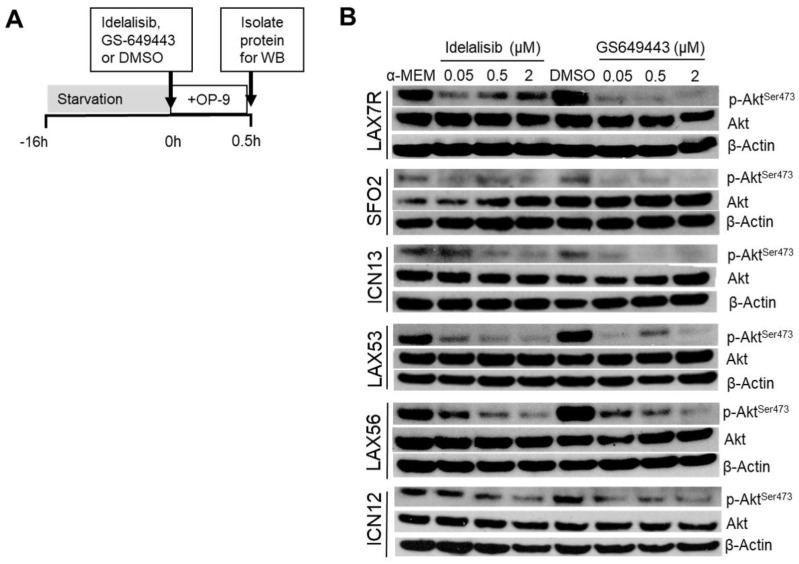
Treatment with the PI3Kδ inhibitors idelalisib and GS-649443 decreases p-Akt levels in B ALL. (**A**) Timeline of starvation/stimulation assay. (**B**) Western blot showing relative phosphorylation of Akt after treatment with either Idelalisib or GS-649443.

These results showed that de novo contact with stromal cells provided a potent activation of the Akt pathway. We next tested if idelalisib or GS-649443 could inhibit Akt phosphorylation mediated by such contact (Figure 3A). ALL cells tested were stimulated by OP9s to phosphorylate Akt in the presence of media control but when the cells are treated with the PI3Kδ inhibitors idelalisib or GS-649443 they demonstrated inhibition of p-Akt detectable by Western blot (Figure 3B), with some samples such as LAX7R and LAX53 showing significant inhibition even at the lowest dose tested, whereas other samples such as ICN13 and ICN12 requiring higher drug concentrations. We conclude that the effect of idelalisib and GS-649443 on p-Akt levels in the leukemia samples is consistent with effective inhibition of the PI3K/Akt pathway.

### 2.4. Idelalisib Inhibits Migration of ALL Cells to SDF-1α

**Figure 4:**
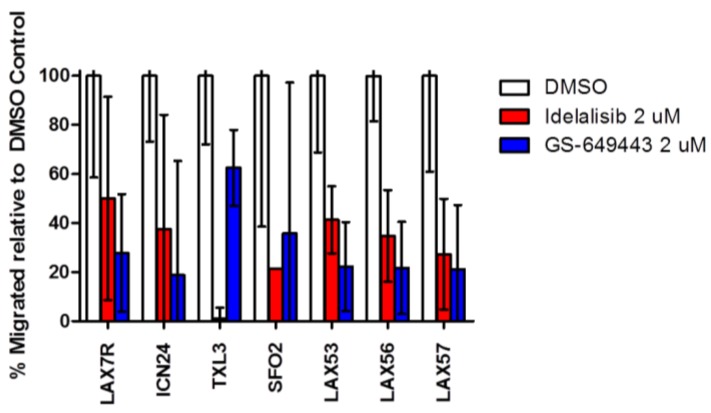
Treatment with PI3Kδ inhibitors idelalisib and GS-649443 decreases migration toward SDF-1α. These are the results of a transwell migration assay wherein ALL cells move across a porous membrane towards SDF-1α (500 ng/mL). We compared the percentage of migrated ALL cells treated with Idelalisib or GS-649443 to DMSO control. Each condition was run in triplicate and the mean of triplicates ±95% CI is graphed above. All treated samples show significant decrease (*p* < 0.001) in migration compared to untreated samples, except TXL3 with GS-649443 which was significant to *p* < 0.01.

Seven of the above ten primary patient samples were plated in the top of a transwell with SDF-1α in the bottom. As expected and reported previously [17], ALL cells have significant migration toward SDF-1α compared to medium control (Supplemental Figure S1). We observed that treatment with either idelalisib or GS-649443 inhibited migration to SDF-1α (500 ng/mL) when compared to DMSO control in all primary samples tested (Figure 4). Inhibition of migration was similar after either idelalisib or GS-649443 treatment. Treatment with lower concentrations of idelalisib (0.1, 0.5, and 2 μM) also decreased migration towards lower doses of SDF-1α (200 ng/mL) (Supplemental Figure S2).

### 2.5. Idelalisib Inhibits Homing of ALL Cells to the Bone Marrow

**Figure 5:**
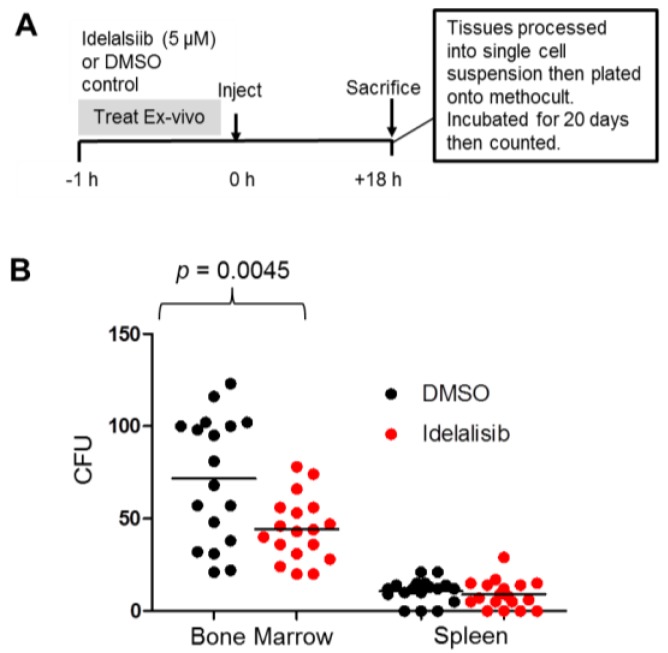
Inhibition of ALL homing to bone marrow after treatment of cells with idelalisib. (**A**) Timeline of experiment. (**B**) Scatterplot of CFUs of LAX56 cells recovered from tissues of mice after ex vivo treatment with idelalisib or DMSO control. Each dot represents the number of CFUs counted on a single plate that was seeded with 5 × 10^4^ mononuclear cells from the tissue specified.

The observation that idelalisib inhibited the migration of ALL cells toward SDF-1α suggested that idelalisib may inhibit the homing of ALL cells to the SDF-1α-rich bone marrow niche. To test this possibility, we injected ALL cells (sample LAX56) into NSG mice after incubating the cells in vitro with either idelalisib (treatment group) or DMSO (control) prior to injection. Eighteen hours post-injection the mice were sacrificed, their spleens and bone marrows were harvested and were processed into a single cell suspension, respectively. The cell suspensions then underwent red blood cell lysis. A standardized number of the remaining mononuclear cells were plated onto medium. Plates that contained cells from the bone marrows of mice in the treatment groups had fewer colony forming units (CFUs) of ALL cells compared to DMSO control. There was no significant difference in CFUs recovered from the spleen between the two groups. The highly significant difference in CFUs recovered from the bone marrow of mice in the two groups (Figure 5) implies that the pretreatment with idelalisib affects the ability of these ALL cells to home to the bone marrow. There did not seem to be a difference in CFUs derived from spleen cells, implying there may be a mechanism by which ALL cells get there distinct from that for bone marrow.

### 2.6. Effect of Idelalisib on Proliferation of ALL Cells

**Figure 6:**
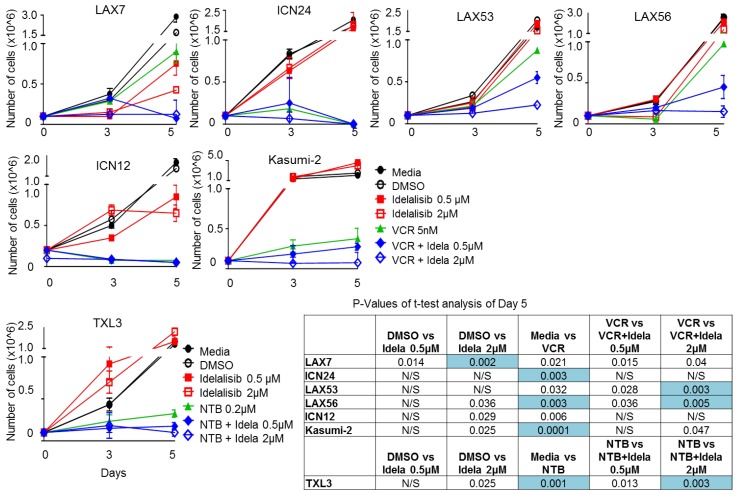
Effect of different concentrations of idelalisib (Idela) on proliferation of ALL cells alone and in combination with Vincristine (5 nM). The mean of three counts under trypan blue exclusion is graphed ± SD for days 3 and 5, day 0 is plotted based on amount of cells first plated. Additionally, the inset is a table of *p* values from the *t*-test comparisons of results of different conditions at day 5. *p* values > 0.05 are listed as not significant (N/S). *p* values ≤ 0.005 are highlighted to show they meet our cutoff for statistical significance.

Six ALL samples were plated in the presence of either Vincristine (VCR), idelalisib, a combination of both, or DMSO control. A seventh ALL sample, TXL3, was plated with the same conditions except using nilotinib instead of VCR. Idelalisib monotherapy did not consistently decrease proliferation at either day 3 or day 5 (Figure 6). VCR (or Nilotinib for BCR-ABL1 positive TXL3) reduced proliferation of ALL cells relative to media control in five of seven samples tested. Three of the seven samples (LAX53, LAX56, and TXL3) showed a further decrease in proliferation on day 5 with combination therapy when compared to VCR 5 nM (or Nilotinib for BCR-ABL1 positive TXL3) monotherapy, with no difference noted on day 3.

Five ALL samples were then tested with idelalisib 2 µM, VCR (either 0.5 nM, 1 nM, or 5 nM, a combination of idelalisib and VCR, or DMSO control. Vincristine decreased proliferation of all five samples tested (Supplemental Figure S3) at all concentrations with most samples showing a dose-dependent decrease in proliferation. We looked for an additive effect of idelalisib in addition to VCR by comparing the same VCR concentration with and without idelalisib. We saw a significant decrease in proliferation due to idelalisib 2 µM with VCR 0.5 nM in Kasumi2 and with VCR 5 nM with LAX57; the other conditions tested and other samples tested did not show any statistically significant change in proliferation. Notably, the differences in proliferation at five days with LAX53 and LAX56 did not reach the threshold of significance in this experiment at 5 nM of VCR and 2 µM of idelalisib, whereas they did in the prior experiment (Figure 6).

## 3. Discussion

We have found that PI3Kδ is expressed in all thirteen tested B ALL samples with various genetic lesions, as were the isoforms PI3Kα, β, and γ (Figure 1). We have shown that these B ALL cells increase phosphorylation of Akt upon stromal contact (Figure 2) and that treatment with idelalisib or GS-649443, both PI3Kδ inhibitors, inhibits this (Figure 3). In contrast to the other published experiments with B ALL showing apoptosis and decreased proliferation with monotherapy [13,14], we could only determine decreased proliferation in a subset of B ALL cases with combination therapy (Figure 6 and Supplemental Figure S3). This difference could be explained by the fact that we tested different patient samples than were in either of the prior reports, but may also have to do with differences in experimental setup. For instance, one of the referenced experiments [14] uses smaller wells without stroma, a different medium, a different viability assay, and their cells are fresh from the patient whereas ours are passaged through a mouse and stored frozen prior to the experiment. There was no robust effect of idelalisib on proliferation either in monotherapy or in the combinations attempted, although there was a small decrease in proliferation at five days in a subset of cells, suggesting a possible effect of idelalisib on a subset of ALL. No immediate relation to karyotype, immunophenotype, or relapse status is evident in the pattern of responses to therapy. Further analysis in a larger number of primary ALL samples may determine if idelalisib inhibits proliferation of a subset of ALL.

We have shown that idelalisib and GS-649443 inhibit migration in vitro consistently across a diverse panel of leukemia samples (Figure 4). Idelalisib also inhibits homing of ALL cells in vivo in a mouse model (Figure 5), an effect that is consistent with those seen in CLL [19,20] but that has not previously been demonstrated in ALL. Fewer CFUs were seen growing from bone marrow from mice in the treatment group than the control group; this difference cannot be explained by decreased survival of the leukemic cells because we did not observe apoptosis after idelalisib monotherapy in vitro [22].

The effects of Idelalisib on cell migration can be mediated both by Akt-dependent and independent mechanisms. PI3K activity is responsible for the generation of phosphatidylinositol (3, 4, 5)-trisphosphate (PIP3) from PIP2. PIP3 can recruit exchange factors for Rac GTPases to the plasma membrane where they can be activated and generate GTP-bound Rac. This in turn will regulate actin cytoskeletal rearrangements needed for cell motility. However, PIP3 also allows docking of Akt at the plasma membrane where it can be activated. As activated Akt is known to phosphorylate proteins involved in regulation of the actin cytoskeleton [23], reduction in levels of pAkt can inhibit motility of cells as well.

PI3Kδ is crucial for normal B cell motility [24] and our data and others suggest that a variety of B ALL types also require it for motility. What remains to be seen is if this impairment of homing will be useful in treating resistant leukemia. Perhaps inhibition of homing of circulating ALL cells to the bone marrow or even mobilization of leukemia cells from their protective niches in the bone marrow can interfere with CAM-DR. This mechanism would be especially appealing for targeting minimal residual disease, where it is possible that minor clones with resistance have now been selected for. Further pre-clinical evaluation of idelalisib is warranted in the hopes of finding a promising combination that can be considered for clinical trials.

## 4. Materials and Methods

### 4.1. Cell Culture

Primary B ALL cells were plated on tissue culture plates with irradiated murine calvarium-derived mesenchymal stromal (OP9) cells in α-MEM with 20% fetal bovine serum (Invitrogen-Thermo Fisher Scientific, Waltham, MA, USA) and 100 U/mL penicillin, 100 μg/mL streptomycin (Invitrogen) (complete media). Established B ALL cell lines (Bel-1 and Kasumi-2) were grown in suspension using RPMI medium with 10% fetal bovine serum and with medium refreshed at least weekly.

### 4.2. PI3K Isoforms

B ALL cells were harvested from co-culture and washed with Dulbecco’s Phosphate Buffered Saline (DPBS). OP9 cells were detached with trypsin and washed with DPBS. Protein was isolated from B ALL cells and OP9s using mammalian protein extraction reagent (ThermoFisher Scientific) with Halt Protease Inhibitor Cocktail (ThermoFisher Scientific) and with mechanical lysis. Protein concentrations were determined and 25 µg of each protein was loaded into wells of SDS-PAA gels (Invitrogen). Separate gels were run for each isoform. Western blotting was performed for PI3Kα, PI3Kβ, PI3Kδ, and PI3Kγ using corresponding antibodies (Santa Cruz Biotechnology, Dallas, TX, USA). β-actin (Santa Cruz Biotechnology) was used as a loading control.

### 4.3. p-Akt^ser473^ Activation/Inhibition

For p-Akt activation, ALL cells were serum starved for 4 h, and plated on plates coated with various ligands (human fibronectin, laminin, VCAM-1) or OP9 stromal cells for a one-hour activation period, prior to protein isolation. BSA was used as a control for the ligands and for the stromal cells our control was a well with typical growth medium (RPMI or α-MEM) without added FBS. As an additional control to separate contact stimulation by stromal cells from stimulation by soluble factors, we also plated ALL cells separated by a transwell membrane (5 μm pore size) from the stromal cells. For p-Akt inhibition, ALL cells were serum-starved overnight and then pretreated for 10 min with either idelalisib or GS-649443 at concentrations of 0.05 μM, 0.5 μM, and 2 μM. The cells with drug were then plated onto OP9 cells. Proteins were isolated after 30 min as described above for Western blot for p-Akt^ser473^ (Cell Signaling Technology, Danvers, MA, USA), total Akt (Cell Signaling Technology), and β-actin (Santa Cruz Biotechnology).

### 4.4. Migration Assay

Leukemia cells were washed with DPBS, then suspended in α-MEM with idelalisib or GS-649443 or a DMSO control. ALL cells were transferred to the upper chamber of a transwell plate (Corning, Corning, NY, USA) while the lower chamber contained α-MEM alone or with 500 ng or 200 ng/mL SDF-1α. After overnight incubation, the numbers of the cells in the lower chamber were counted in a hemocytometer using trypan blue exclusion.

### 4.5. Homing and Colony Forming Assay

LAX56 cells were incubated with either idelalisib 5 µM or DMSO as a vehicle control for one hour. The 5 µM concentration used is similar to the Cmax of idelalisib in human trials at tolerated doses [25]. The cells were washed and injected into the tail vein of NOD.Cg-Prkdc^scid^Il2rg^tm1Wjl^/SzJ (NSG) mice (5 × 10^6^ cells/mouse, n = 6/group). Eighteen hours after injection the mice were euthanized using CO2. Their spleen, bone marrow, blood, and lungs were collected and processed into cell suspensions separately and underwent red cell lysis, then plated in a cell culture dish (ThermoFisher Scientific) coated with methylcellulose-based medium with recombinant human cytokines (Methocult Enriched; Stem Cell Technologies, Vancouver, BC Canada) per the manufacturer’s instructions. 5 × 10^4^ mononuclear cells/dish were plated in triplicate for each organ checked in each mouse. Plates were monitored until sufficient colonies were present to count 20 days later. Colonies were defined as accumulations of >30 cells.

### 4.6. Proliferation Assay

Leukemia cells (0.5 × 10^6^) were treated with DMSO, idelalisib, Vincristine, or a combination of vincristine and idelalisib in 1 mL of complete media on 24-well tissue culture plates coated with irradiated OP9. For the sample with a BCR-ABL1 translocation nilotinib 0.2 µM was used in place of VCR. One experiment (Figure 6) was set up with a single dose of VCR (5 nM) and varying doses of idelalisib (0.5 µM, 2 µM) whereas another experiment (Figure S2) was set up with one dose of idelalisib (2 µM) and varying doses of VCR (0.5 nM, 1 nM, or 5 nM). The cells were counted by trypan blue exclusion to measure proliferation on days 3 and 5. In the figure we list all *p* values < 0.05 but due to the high number of comparisons within the experiment we are only considering a comparison statistically significant if the *p* value is ≤0.005.

### 4.7. Statistical Analysis

All graphs and statistics calculations were carried out using Graphpad Prism. *p* values were determined by Student’s *t*-test. *p* values less than 0.05 were used by convention for statistical significance, except in analysis of the proliferation assay, where *p* < 0.005 was used as a more robust standard due to the multiple comparisons. In the *p* values in the figure we still listed *p* values < 0.05, but in the analysis we did not consider these significant.

## 5. Conclusions

Idelalisib and GS-649443 both consistently decrease p-Akt and migration towards SDF-1α in a diverse sample of B ALL. Idelalisib pretreatment causes diminished homing of ALL to bone marrow in mice. Idelalisib may decrease proliferation in a subset of evaluated ALL cells when combined with vincristine. More pre-clinical studies are required to see if clinical trials of idelalisib in B ALL are warranted.

## Supplementary Materials

The following are available online at http://www.mdpi.com/2072-6694/9/9/121/s1; Supplementary Table S1: Characteristics of Human ALL cells. Figure S1: ALL cells migrate less to medium control than to SDF-1α and this migration is unaffected by PI3Kδ inhibitors. Figure S2: Treatment with PI3Kδ inhibitors idelalisib decreases migration toward SDF-1α (200 ng/mL). Figure S3: Effect of idelalisib on proliferation of ALL cells alone and in combination with different concentrations of Vincristine.

## References

[1] Hunger S.P., Lu X., Devidas M., Camitta B.M., Gaynon P.S., Winick N.J., Reaman G.H., Carroll W.L. (2012). Improved survival for children and adolescents with acute lymphoblastic leukemia between 1990 and 2005: A report from the children’s oncology group. J. Clin. Oncol..

[2] Bhojwani D., Pui C.H. (2013). Relapsed childhood acute lymphoblastic leukaemia. Lancet Oncol..

[3] Shishido S., Bonig H., Kim Y.M. (2014). Role of integrin alpha4 in drug resistance of leukemia. Front. Oncol..

[4] Vivanco I., Sawyers C.L. (2002). The phosphatidylinositol 3-kinase akt pathway in human cancer. Nat. Rev. Cancer.

[5] Chantry D., Vojtek A., Kashishian A., Holtzman D.A., Wood C., Gray P.W., Cooper J.A., Hoekstra M.F. (1997). P110delta, a novel phosphatidylinositol 3-kinase catalytic subunit that associates with p85 and is expressed predominantly in leukocytes. J. Biol. Chem..

[6] Jou S.T., Carpino N., Takahashi Y., Piekorz R., Chao J.R., Carpino N., Wang D., Ihle J.N. (2002). Essential, nonredundant role for the phosphoinositide 3-kinase p110delta in signaling by the b-cell receptor complex. Mol. Cell. Biol..

[7] Vanhaesebroeck B., Khwaja A. (2014). PI3Kδ inhibition hits a sensitive spot in b cell malignancies. Cancer Cell..

[8] Rodon J., Dienstmann R., Serra V., Tabernero J. (2013). Development of PI3K inhibitors: Lessons learned from early clinical trials. Nat. Rev. Clin. Oncol..

[9] Janne P.A., Gray N., Settleman J. (2009). Factors underlying sensitivity of cancers to small-molecule kinase inhibitors. Nat. Rev. Drug Discov..

[10] Burger J.A., Okkenhaug K. (2014). Haematological cancer: Idelalisib-targeting PI3Kδ in patients with b-cell malignancies. Nat. Rev. Clin. Oncol..

[11] Furman R.R., Sharman J.P., Coutre S.E., Cheson B.D., Pagel J.M., Hillmen P., Barrientos J.C., Zelenetz A.D., Kipps T.J., Flinn I. (2014). Idelalisib and rituximab in relapsed chronic lymphocytic leukemia. N. Engl. J. Med..

[12] Tasian S.K., Teachey D.T., Li Y., Shen F., Harvey R.C., Chen I.M., Ryan T., Vincent T.L., Willman C.L., Perl A.E. (2017). Potent efficacy of combined PI3K/mTOR and JAK or ABL inhibition in murine xenograft models of ph-like acute lymphoblastic leukemia. Blood.

[13] Rosin N.Y., Koehrer S., Kim E., O’Brien S., Wierda W.G., Thomas D.A., Estrov Z., Kantarjian H.M., Lannutti B.J., Burger J.A. (2012). In vitro effects of pi3k delta inhibitor gs-1101 (Cal-101) in acute lymphoblastic leukemia (ALL). Blood.

[14] Eldfors S., Kuusanmaki H., Kontro M., Majumder M.M., Parsons A., Edgren H., Pemovska T., Kallioniemi O., Wennerberg K., Gokbuget N. (2017). Idelalisib sensitivity and mechanisms of disease progression in relapsed TCF3-PBX1 acute lymphoblastic leukemia. Leukemia.

[15] Patel L., Chandrasekhar J., Evarts J., Forseth K., Haran A.C., Ip C., Kashishian A., Kim M., Koditek D., Koppenol S. (2016). Discovery of orally efficacious phosphoinositide 3-kinase delta inhibitors with improved metabolic stability. J. Med. Chem..

[16] Yahiaoui A., Meadows S.A., Sorensen R.A., Cui Z.H., Keegan K.S., Brockett R., Chen G., Quéva C., Li L., Tannheimer S.L. (2017). PI3Kδ inhibitor idelalisib in combination with BTK inhibitor ono/gs-4059 in diffuse large b cell lymphoma with acquired resistance to PI3Kδ and btk inhibitors. PLoS ONE.

[17] Yao H., Price T., Olivere L., Warner M., Tannheimer S., Sipkins D.A. (2016). PI3K delta inhibition suppresses central nervous system involvement of acute lymphoblastic leukemia. Blood.

[18] Thomas X., Anglaret B., Bailly M., Maritaz O., Magaud J.P., Archimbaud E. (1998). Differential adhesiveness between blood and marrow leukemic cells having similar pattern of vla adhesion molecule expression. Leuk. Res..

[19] Hoellenriegel J., Meadows S.A., Sivina M., Wierda W.G., Kantarjian H., Keating M.J., Giese N., O’Brien S., Yu A., Miller L.L. (2011). The phosphoinositide 3’-kinase delta inhibitor, cal-101, inhibits b-cell receptor signaling and chemokine networks in chronic lymphocytic leukemia. Blood.

[20] Fiorcari S., Brown W.S., McIntyre B.W., Estrov Z., Maffei R., O’Brien S., Sivina M., Hoellenriegel J., Wierda W.G., Keating M.J. (2013). The PI3-kinase delta inhibitor idelalisib (GS-1101) targets integrin-mediated adhesion of chronic lymphocytic leukemia (CLL) cell to endothelial and marrow stromal cells. PLoS ONE.

[21] Lannutti B.J., Meadows S.A., Herman S.E., Kashishian A., Steiner B., Johnson A.J., Byrd J.C., Tyner J.W., Loriaux M.M., Deininger M. (2011). Cal-101, a p110delta selective phosphatidylinositol-3-kinase inhibitor for the treatment of b-cell malignancies, inhibits pi3k signaling and cellular viability. Blood.

[22] Adam E., Kim H.N., Gang E.J., Schnair C., Lee S., Lee S., Khazal S., Kosoyan O., Konopleva M., Parekh C. (2017). Department of Pediatrics, Division of Hematology, Oncology and Blood and Marrow Transplantation, Children’s Hospital Los Angeles, University of Southern California Keck School of Medicine, Los Angeles, CA, USA.

[23] Xue G., Hemmings B.A. (2013). PKB/Akt-dependent regulation of cell motility. J. Natl. Cancer Inst..

[24] Reif K., Okkenhaug K., Sasaki T., Penninger J.M., Vanhaesebroeck B., Cyster J.G. (2004). Cutting edge: Differential roles for phosphoinositide 3-kinases, p110gamma and p110delta, in lymphocyte chemotaxis and homing. J. Immunol..

[25] Brown J.R., Byrd J.C., Coutre S.E., Benson D.M., Flinn I.W., Wagner-Johnston N.D., Spurgeon S.E., Kahl B.S., Bello C., Webb H.K. (2014). Idelalisib, an inhibitor of phosphatidylinositol 3-kinase p110delta, for relapsed/refractory chronic lymphocytic leukemia. Blood.

