# Supplementary material for: The PI3Kδ Inhibitor Idelalisib Inhibits Homing in an in Vitro and in Vivo Model of B ALL
Source: Cancers (Basel). 2017 Sep 10;9(9):121. doi: 10.3390/cancers9090121 (PMC5615336; doi:10.3390/cancers9090121)
Supplement: Supplementary file 1 [file cancers-09-00121-s001.pdf]

Supplementary Table S1: Characteristics of Human ALL cells

Human leukemia ID	Immuno-phenotype	Sample type	Cytogenetics
**LAX7**	Pre B ALL	Diagnosis	Normal
**LAX7R**	Pre B ALL	Relapse of LAX7	KRASG12V
**ICN24**	Pre B ALL	Diagnosis	Unknown
**LAX53**	Pre B ALL	Diagnosis	Unknown
**LAX56**	Pre B ALL	Relapse	t(Y;7)(p1.3;p13)
**LAX57**	Pre B ALL	Diagnosis	t(1;9)(q44;p22)
**TXL3**	Pre B ALL	Diagnosis	BCR-ABL1 p210
**SFO2**	Pre B ALL	Relapse	BCR-ABL1 p210
**ICN12**	Pre B ALL	Diagnosis	E2A-PBX1
**ICN13**	Pro B ALL	Diagnosis	MLL-AF4 t(4;11)(q21;q23)
**BEL-1**	Pro B ALL	Diagnosis	MLL-AF4 t(4;11)(q21;q23)
**RS4;11**	Pro B ALL	Diagnosis	MLL-AF4 t(4;11)(q21;q23)
**Kasumi-2**	Pre B ALL	Diagnosis	E2A-PBX1

S1. ALL cells migrate less to medium control than to SDF-1α and this migration is unaffected by PI3Kδ inhibitors. Transwell migration assay of ALL cells treated with a PI3Kδ inhibitor compared to DMSO control, all without a chemoattractant. This experiment was run in parallel to the one with SDF-1α and serves as a control – the percentages graphed are all relative to the DMSO control migration towards SDF-1α from Figure 4. Mean of triplicates +/- 95% CI. There is no significant difference in migration using this assay between treated and untreated samples for ALL cells without SDF-1α.

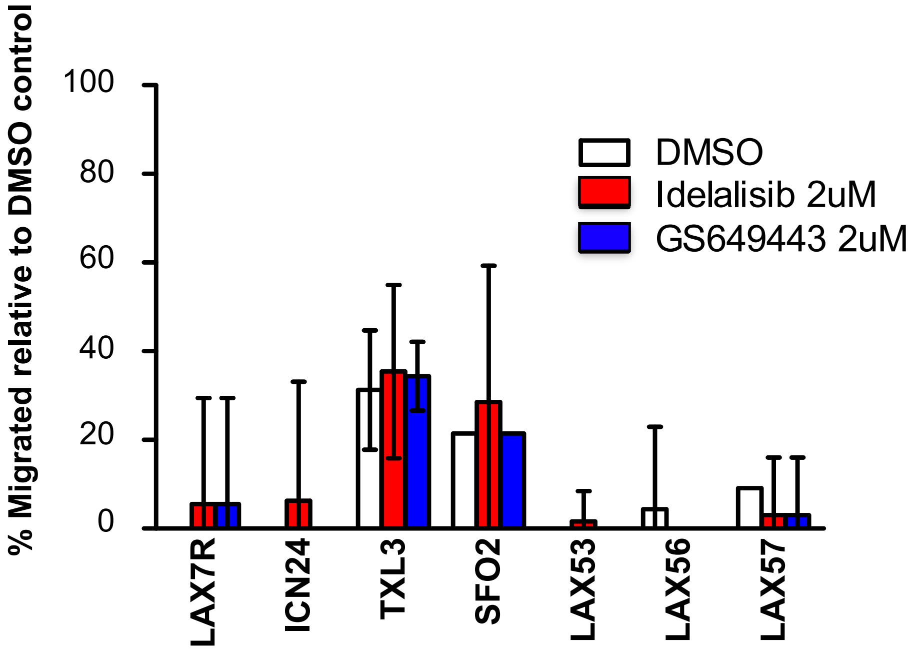

S2. Treatment with PI3Kδ inhibitors idelalisib decreases migration toward SDF-1α (200ng/ml). Transwell migration assay of ALL cells towards SDF-1α **(200ng/ml)** comparing cells treated with different doses of idelalisib (0.1 μM, 0.5 μM and 2 μM) to DMSO control. Mean of triplicates +/- 95% CI. All samples treated with 0.5μM and 2μM idelalisib show significant decrease in migration compared to untreated samples (*= p<0.05, **=p<0.001, ***=p<0.005).

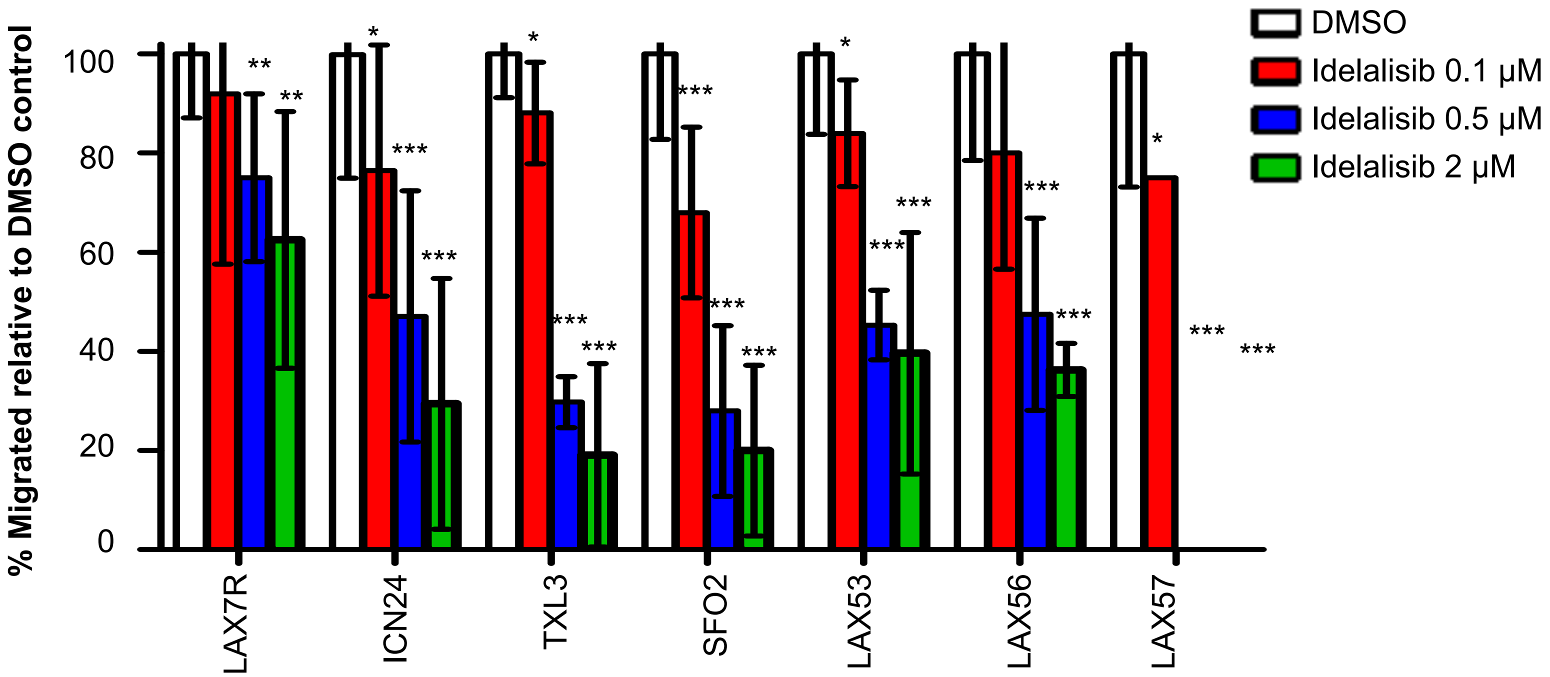

S3. Effect of Idelalisib on proliferation of ALL cells alone and in combination with different concentrations of Vincristine. Mean of 3 counts under trypan blue exclusion is graphed +/- SD for days 3 and 5, day 0 is plotted based on amount of cells first plated. 3 different doses of Vincristine (VCR) (0.5, 1 and 5 nM) were tested. Idelalisib (Idela) concentration is 2μM in all instances below where it is used. Also inset is a table of p-values from t-test comparisons of results of different conditions at Day 5. P-values >0.05 are listed as not significant (N/S).

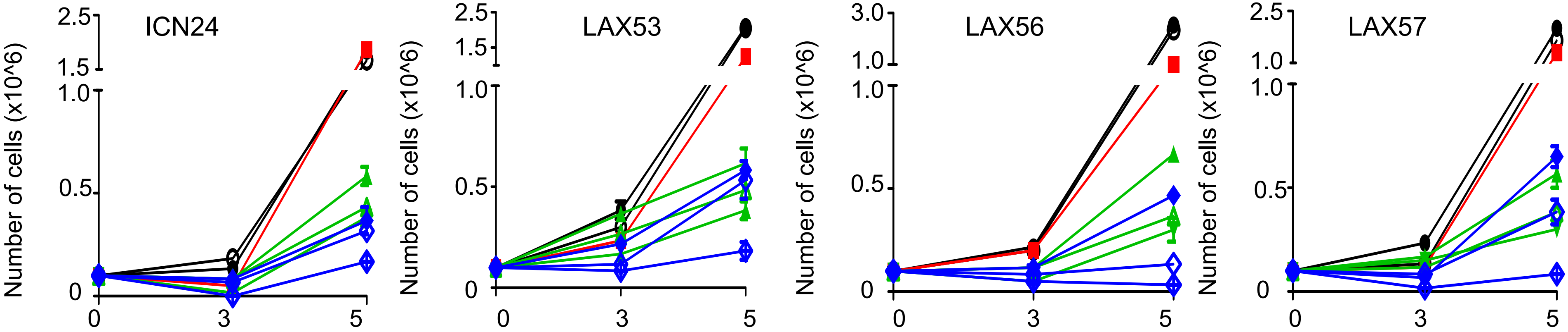

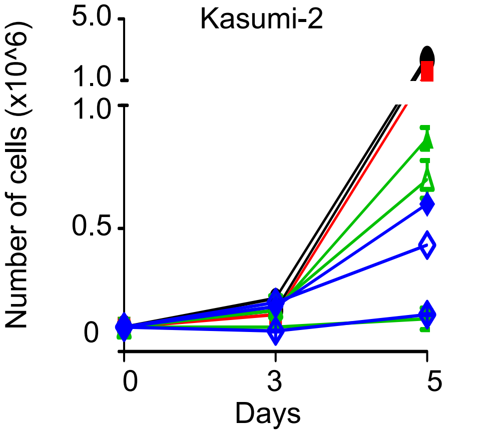

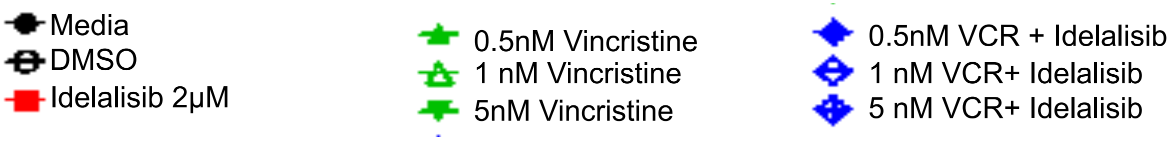

P-Values of t-test analysis of Day 5

	Media vs 0.5nM VCR	Media vs 1nM VCR	Media vs 5nM VCR	DMSO vs Idela	0.5nM VCR vs 0.5nM VCR + Idela	1nM VCR vs 1nM VCR+ Idela	5nM VCR vs 5nM VCR + Idela
**ICN24**	0.0042	0.0021	0.002	N/S	N/S	0.0078	0.01
**LAX53**	0.0009	0.0004	0.0004	0.0085	N/S	N/S	0.0327
**LAX56**	5.03E-06	2.87E-06	6.65E-06	0.0075	0.0132	0.0078	0.0161
**LAX57**	0.0024	0.0013	0.0001	N/S	N/S	N/S	0.0002
**Kasumi-2**	0.0005	0.0004	0.0002	N/S	0.0003	0.027	N/S
